# Sequential Circulating Tumor Cell Counts in Patients with Locally Advanced or Metastatic Hepatocellular Carcinoma: Monitoring the Treatment Response

**DOI:** 10.3390/jcm9010188

**Published:** 2020-01-10

**Authors:** Kun-Ming Rau, Chien-Ting Liu, Yu-Chiao Hsiao, Kai-Yin Hsiao, Tzu-Min Wang, Wei-Shan Hung, Yu-Li Su, Wei-Ching Liu, Cheng-Hsu Wang, Hsueh-Ling Hsu, Po-Heng Chuang, Ju-Chien Cheng, Ching-Ping Tseng

**Affiliations:** 1 Department of Hematology-Oncology, E-Da Cancer Hospital, Kaohsiung 824, Taiwan; liu07822@ms57.hinet.net; 2 School of Medicine, College of Medicine, I-Shou University, Kaohsiung 824, Taiwan; 3 Division of Hematology-Oncology, Department of Internal Medicine, Kaohsiung Chang Gung Memorial Hospital, Kaohsiung 833, Taiwan; m7155@cgmh.org.tw (C.-T.L.); kilu9818@gmail.com (K.-Y.H.); yolisu@mac.com (Y.-L.S.); a0920365248@gmail.com (W.-C.L.); 4 College of Medicine, Chang Gung University, Taoyuan 333, Taiwan; chwang@adm.cgmh.org.tw; 5 Department of Medical Biotechnology and Laboratory Science, College of Medicine, Chang Gung University, Taoyuan 333, Taiwan; lian31602@hotmail.com (Y.-C.H.); sugarcane025017@gmail.com (T.-M.W.); srhhung@gmail.com (W.-S.H.); snow_0502@hotmail.com (H.-L.H.); 6 Division of Hematology/Oncology, Department of Internal Medicine, Keelung Chang Gung Memorial Hospital, Keelung 204, Taiwan; 7 Department of Internal Medicine, China Medical University Hospital, Taichung 404, Taiwan; poheng2@yahoo.com.tw; 8 Department of Medical Laboratory Science and Biotechnology, China Medical University, Taichung 404, Taiwan; jccheng@mail.cmu.edu.tw; 9 Graduate Institute of Biomedical Sciences, College of Medicine, Chang Gung University, Taoyuan 333, Taiwan; 10 Molecular Medicine Research Center, Chang Gung University, Taoyuan 333, Taiwan; 11 Department of Laboratory Medicine, Linko Chang Gung Memorial Hospital, Taoyuan 333, Taiwan

**Keywords:** circulating tumor cells, hepatocellular carcinoma, longitudinal follow-up, alpha-fetoprotein

## Abstract

Hepatocellular carcinoma (HCC) is among the most common causes of cancer death in men. Whether or not a longitudinal follow-up of circulating tumor cells (CTCs) before and at different time points during systemic/targeted therapy is useful for monitoring the treatment response of patients with locally advanced or metastatic HCC has been evaluated in this study. Blood samples (*n* = 104) were obtained from patients with locally advanced or metastatic HCC (*n* = 30) for the enrichment of CTCs by a negative selection method. Analysis of the blood samples from patients with defined disease status (*n* = 81) revealed that those with progressive disease (PD, *n* = 37) had significantly higher CTC counts compared to those with a partial response (PR) or stable disease (SD; *n* = 44 for PR + SD, *p* = 0.0002). The median CTC count for patients with PD and for patients with PR and SD was 50 (interquartile range 21–139) and 15 (interquartile range 4–41) cells/mL of blood, respectively. A longitudinal analysis of patients (*n* = 17) after a series of blood collections demonstrated that a change in the CTC count correlated with the patient treatment response in most of the cases and was particularly useful for monitoring patients without elevated serum alpha-fetoprotein (AFP) levels. Sequential CTC enumeration during treatment can supplement standard medical tests and benefit the management of patients with locally advanced or metastatic HCC, in particular for the AFP-low cases.

## 1. Introduction

The absolute incidence and mortality of hepatocellular carcinoma (HCC) worldwide reported in 2018 was 841,080 and 781,631, respectively [1]. It is the second most common cancer and the most common cause of cancer-related deaths in men in Taiwan. Hepatitis B virus (HBV)/hepatitis C virus (HCV) infection and alcohol abuse are among the major etiologies for liver diseases and cancer. The underlying etiology of HCC due to HBV and HCV infections in Taiwan has been reduced because of universal HBV vaccination and the recent development of highly effective anti-HCV agents [2,3]. However, there is a parallel increase in HCC related to nonalcoholic fatty liver disease [4].

Once HCC is diagnosed, the Barcelona Clinic Liver Cancer algorithm, which includes in classifying HCC patients, clinical variables associated with tumor burden, the degree of liver dysfunction, and the patient’s symptoms, is used for disease staging and treatment allocation [5,6]. A number of strategies have been employed for the treatment of patients with HCC. Transplantation remains the best option, but the supply of donor organs is limited. Alternative curative treatments including resection, radiofrequency ablation (RFA) [7], and, potentially, systemic and targeted therapy may delay recurrence [8,9]. When patients experience recurrence following the initial local therapies or transplantation, another local therapy and/or systemic treatments are required to obtain the best possible outcome. Abdominal ultrasound, radiological imaging, and serum alpha-fetoprotein (AFP) levels are the most common methods for surveillance of the treatment response. Imaging by computed tomography (CT) scans exposes patients to radiation, while serum AFP levels are in the normal range in 15–30% of patients with HCC [10]. For patients who are in clinical remission, all of the above methods cannot detect early recurrence.

During cancer progression, the number of circulating tumor cells (CTCs) increases and correlates with tumor mass [11]. Effective therapies that reduce tumor burden usually correlates with a decrease in the number of CTCs. Monitoring the CTC count is valuable in scrutinizing the treatment response of cancer patients and should provide for better patient care and management [12,13,14]. The presence of CTCs in patients with HCC has been reported in several studies [15,16,17]. CTC counts have been analyzed in most of these studies at a specific time point without longitudinal follow-up concerning the number or the change in CTC counts. Whether or not serial CTC counts during the course of treatment provides an advantage for monitoring the treatment response and disease status of patients with HCC remains to be explored.

In this study, patients with locally advanced or distant metastatic HCC were recruited for a series of CTC enumeration using a negative selection platform PowerMag which allows efficient depletion of CD45^+^ leukocytes and isolation of viable and label-free CTCs even only one CTC is present in 4 mL of peripheral blood [18]. The CD45^+^ leukocytes are depleted for 10–20 folds more efficient by the PowerMag platform than by the EasySep method (StemCell Technologies, Vancouver, BC, Canada). The recovery rates of CTCs are 77–82% and 46–62% when cancer cells were enriched from leukocyte suspension and whole blood, respectively. The number of cancer cells recovered by PowerMag was linearly correlated with the number of cancer cells in the blood [18]. By the PowerMag platform, the number of CTCs and the change in the CTC counts were analyzed and correlated with the disease status and treatment response of the patients. Performing a series of CTC enumeration in parallel with the current standard medical methods provide benefits for the clinical care of patients with locally advanced or metastatic HCC, in particular for patients without elevated serum AFP levels.

## 2. Experimental Section

### 2.1. Patients

This study was approved by the Institutional Review Board of Chang Gung Memorial Hospital (approval ID: 104-9667B) and E-Da Hospital (approval ID: EMRP66107). The selection criteria of patients were (1) presence of histologically/cytologically or clinically documented diagnosis of locally advanced or metastatic HCC with either serum AFPs ≧400 ng/mL, presence of cirrhosis and/or history of chronic HBV or HCV infections, or morphological evidence such as characteristic hypervascular liver tumors on contrast CT scans or magnetic resonance imaging (MRI); (2) age was >20 years old; (3) advanced HCC, when the patient was ineligible or when the patient did not consent to receiving local invasive treatment (resection, chemoembolization, or RFA); (4) the presence of at least one measurable disease which was defined as a lesion that can be measured as 10 mm in at least 1 dimension with a spiral CT scan or MRI; (5) patients willingness to either accept intra-arterial chemotherapy (IA-CT), intravenous chemotherapy (IV-CT), or targeted therapy such as sorafenib or other systemic therapy such as thalidomide, for HCC; (6) a life expectancy of at least 12 weeks; and (7) patients willing to provide written informed consent to participate in the study. A total of 30 patients with HCC were prospectively enrolled in the Division of Hematology and Oncology, Department of Internal Medicine, Kaohsiung Chang Gung Memorial Hospital. All patients gave written informed consent before enrollment in the study.

### 2.2. Enrichment and Isolation of CTCs

Blood samples were drawn from eligible patients following designed study protocols at day zero (baseline) and at subsequent periods of time after the start of treatment. A PowerMag negative selection system was used to enrich CTCs as described previously [18,19]. Briefly, fresh whole blood samples (4 mL) were processed by lysis of red blood cells (RBC) with an RBC lysis buffer (0.15 M NH_4_Cl and 10 mM NaHCO_3_) at room temperature. The mixture was then centrifuged (400 g) at 10 °C for 10 min. The cell pellets were washed and subsequently resuspended in 2 mL of culture medium to obtain all nucleated cells. To deplete CD45^+^-leukocytes, a CD45 depletion cocktail (StemCell Technologies, Vancouver, BC, Canada) was mixed with the collected nucleated cells and incubated at room temperature for 15 min to label CD45^+^-leukocytes. The sample was then loaded onto a PowerMag column to separate CD45^+^-leukocytes from the other nucleated cells. The cell filtrate was then centrifuged (400× *g*) for 10 min and the procedure was repeated four times to remove most of the leukocytes. The pelleted cells were subject to further analysis.

### 2.3. Immunofluorescence Staining and CTC Counting

For immunofluorescence staining, CD45^+^-depleted cell filtrates were incubated with an anti-EpCAM antibody (Abcam Inc., Cambridge, England) in the presence of the DNA staining dye Hoechst 33,342 at room temperature in the dark for 1 h as described previously [18,19]. After several washes and centrifugation, the supernatants were removed and the cell pellets were resuspended. The Alexa Fluor 488-conjugated donkey anti-mouse antibody (Invitrogen Inc., Carlsbad, CA, USA) was added to the cell suspension. After incubation in the dark for 30 min, the unbound antibody was removed and the complete cell aliquot was placed on a slide. The full immunofluorescent images were captured by fluorescence microscopy using an automated slide scanning platform (Zeiss Axiovert 200M, Oberkochen, Germany) followed by image analysis using an IN Cell Analyzer 1000 Cellular Imaging and Analysis System (GE Healthcare Life Sciences, Pittsburgh, PA, USA). EpCAM^+^-CTCs were defined as the cells with intact nucleus staining (Hoechst-positive) and positive for EpCAM.

### 2.4. Measurement of Serum AFP

Serum AFP levels were measured at the same time as the CTC counts. An Abbott G4-5539/R05 Kit was used for measuring serum AFP levels. Analytical procedures were performed as described by the manufacturer.

### 2.5. Statistical Analysis

The CTC counts for patients with progressive disease (PD) and patients with partial response (PR) and stable disease (SD) were compared using the Mann–Whitney test. Statistical analysis was performed using Prism 5.0. *p* < 0.05 was considered statistically significant.

## 3. Results

### 3.1. Basic Characteristics of Enrolled Patients

Thirty informative patients with advanced or metastatic HCC were enrolled in this study. The basic characteristics of these patients are shown (Table 1). The median age was 64 (interquartile range 56–69). Of the patients enrolled in this study, 67% and 33% were male and female, respectively. The initial Tumor, Node, Metastasis (TNM) classification of the tumors from 30 patients with HCC was based on the American Joint Committee on Cancer, seventh edition. Eight patients at diagnosis were at stage I, four at stage II, seventeen at stage III, and one at stage IV, respectively. All patients were diagnosed with locally advanced HCC and/or extrahepatic metastasis at the time of enrollment in this study. All clinicopathologic parameters of patients were classified according to the chart records.

Twenty-one patients received sorafenib at enrollment of this study. During the course of treatment, six patients had their treatment changed to thalidomide (*n* = 2), ramucirumab (*n* = 1), sorafenib (*n* = 1), IA-CT (*n* = 1), or IV-CT (*n* = 1). Nine patients who did not take sorafenib were treated with ramucirumab (*n* = 2), nivolumab (*n* = 1), thalidomide (*n* = 2), IA-CT (*n* = 3), or IV-CT (*n* = 1).

Sorafenib was the standard treatment of patients with locally advanced or metastatic HCC in our institute and was prescribed from 400 mg to 800 mg daily based on the performance status and predicted tolerability of patients. The dosage was adjusted according to the side effects of sorafenib, with a maximum dose of 800 mg daily. The regimen for IA-CT included doxorubicin and cisplatin [20]; the regimen for IV-CT was oxaliplatin and fluorouracil [21]. CTC counts every 2–3 months and the measurement of serum AFP levels was performed for monitoring the therapeutic response. Response Evaluation Criteria in Solid Tumors 1.1 (RECIST 1.1) was used for evaluating the therapeutic response according to the imaging study and the serum AFP level [22]. Disease status was defined as PD, SD, and PR, respectively.

### 3.2. CTC Counts are Correlated with Disease Status of Patients with HCC

**Figure 1:**
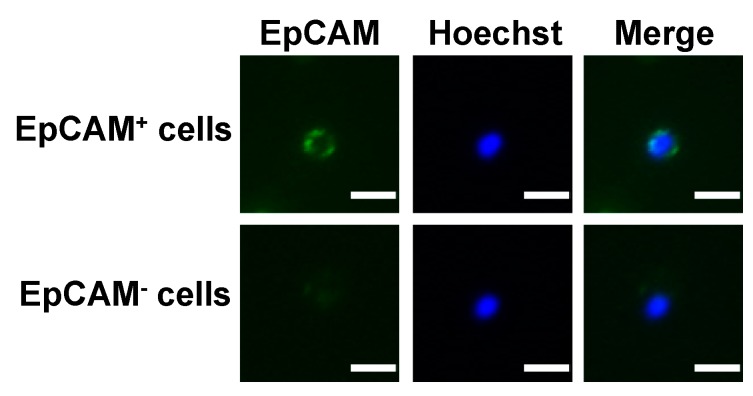
Schematic representation for the fluorescence images of EpCAM^+^ and EpCAM^−^ cells from individuals with advanced or metastatic HCC. The CD45^+^-depleted cell populations that were positive and negative for EpCAM were defined, respectively. Positive staining of Hoechst 33,342 (blue) indicates the presence of intact nucleated cells. Bar =10 µm.

**Figure 2:**
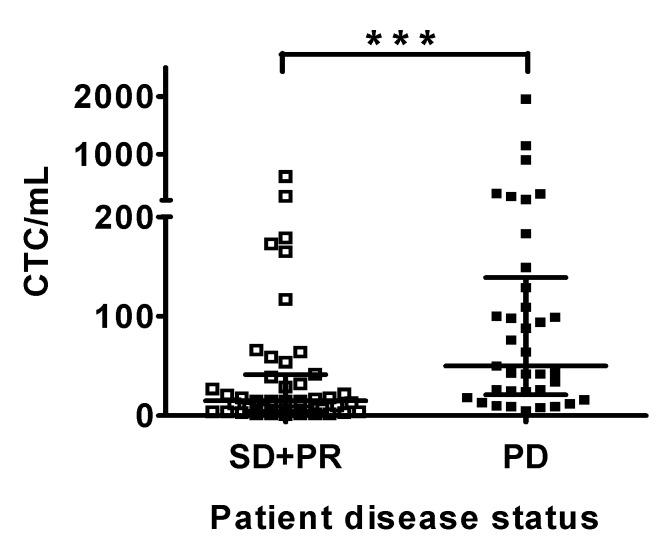
CTC counts for patients with progressive disease (PD), and patients with partial response (PR), or stable disease (SD). The disease status was determined at the time of blood collection for CTC counting. The CTC counts were plotted against the disease status of patients. A significant difference for the CTC counts was observed for the patients at the status of PR + SD and the patients at the status of PD. ***, *p* < 0.0001.

A total of 104 blood samples were collected from patients during the course of chemo- or targeted-therapy (Table 2). The PowerMag negative selection system [18] was used to enrich CD45^−^ cells from peripheral blood by lysis of RBCs and depletion of CD45^+^ leukocytes. CTCs were defined as the EpCAM^−^ positive cells in the CD45^+^-depleted cell filtrates (Figure 1). The disease status for patients at each blood collection was determined and recorded based on an imaging study and the serum AFP level. Whether or not the disease status of patients correlated with the CTC count at that particular time point was analyzed. Thirty-seven occasions for all patients were defined as PD and 44 occasions were defined as PR (*n* = 18) or SD (*n* = 26). CTC counts for patients with PD were significantly higher than patients with PR or SD (*p* = 0.0002). The median CTC count for patients with PD and patients with PR + SD was 50 (interquartile range 21–139) and 15 (interquartile range 4–41) cells/mL of blood, respectively (Figure 2). CTC counts facilitate the differentiation of patients with PD from patients with PR or SD.

### 3.3. Use of CTC Counts for Longitudinal Follow-Up and Disease Monitoring of Patients with HCC

A total of 85 blood samples from 17 patients with at least three blood collections (*n* = 3–9) and two measurements of serum AFP levels (*n* = 2–8) were analyzed for investigating whether or not CTC counts are suitable for disease monitoring of patients with advanced or metastatic HCC. The data are presented in the following sections according to the serum AFP levels. The serum AFP of 100 ng/mL was used as a threshold to divide the patients according to a previous study by Hiraoka et al. who used AFP >100 ng/mL to define positive cases for calculating the tumor marker score as a predictive prognosis value [23].

**Figure 3:**
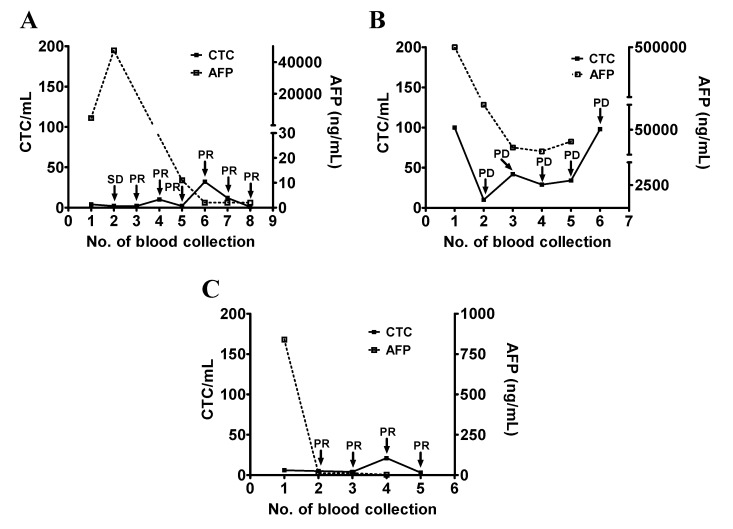
Longitudinal follow-up of the CTC counts for monitoring the disease status of patients with HCC (part I). (**A**–**C**) The blood samples were longitudinally collected from patients #1 (panel **A**), #17 (panel **B**), and #19 (panel **C**), who had a high initial serum AFP level followed by a decrease. Series testing of the CTC count and the serum AFP level were performed and plotted accordingly. The disease status (SD, PR, and PD) was determined and was used as an indicator of the patient’s clinical outcome.

Patients #1, #17, and #19 had an initial serum AFP >100 ng/mL and a decreasing trend in the serum AFP levels during the course of the study. Among these three patients, patient #1 was originally treated with sorafenib and was later changed to thalidomide after the second blood collection due to the side effects of sorafenib. Serum AFP was high at the first and second blood collection (4737 ng/mL and 47,360 ng/mL) when the disease status of the patient was considered as SD and it decreased to normal when the patient was at PR. CTC counts remained mostly low during the study period which was consistent with the clinical status of SD and PR (Figure 3A). Patient #17 had a high serum AFP (>500,000 ng/mL) that decreased during follow-up. CTC counts remained high except for the second collection. The change of the CTC count was consistent with the disease status of the patient (Figure 3B). Patient #19 received ramucirumab and underwent PR. Serum AFP (840 ng/mL) was decreased to normal after a series of treatments, while the CTC counts were low throughout the study (Figure 3C).

**Figure 4:**
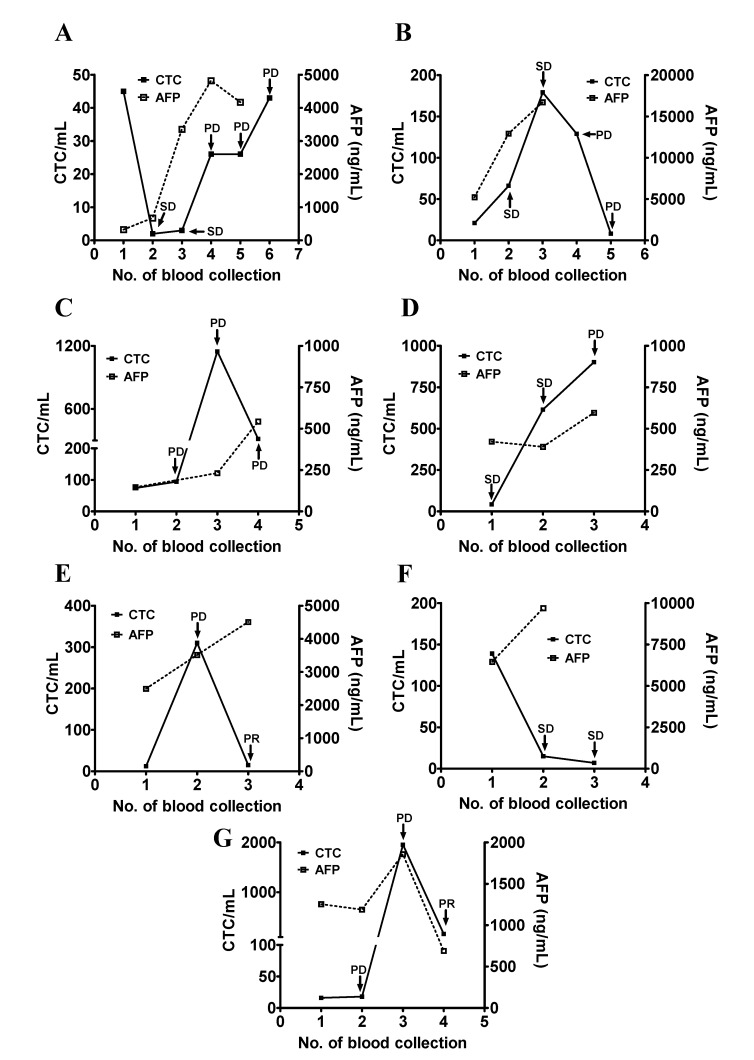
Longitudinal follow-up of the CTC counts for monitoring the disease status of patients with HCC (part II). (**A**–**G**) The blood samples were longitudinally collected from patients #2 (panel **A**), #4 (panel **B**), #10 (panel **C**), #13 (panel **D**), #20 (panel **E**), #22 (panel **F**), and #28 (panel **G**), who all had a high initial serum AFP level followed by an increase. Series testing of the CTC count and serum AFP level were performed and plotted accordingly. The disease status (SD, PR, and PD) was determined and was used as an indicator of the patient’s clinical outcome.

Patients #2, #4, #10, #13, #20, #22, and #28 had an initial serum AFP >100 ng/mL which increased during the course of study. Patient #2 had an initial serum AFP of 323 ng/mL and was treated with sorafenib. The first two treatments appeared to be effective and the patient was at SD during the second and third blood collection. The serum AFP was increased and remained high (675 ng/mL and 3356 ng/mL) which predicted PD at the later time points. CTC counts decreased to below 10 cells/mL and increased as the disease progressed. The CTC count of this patient may be a reasonable marker of this patient’s disease status (Figure 4A). Patient #4 was treated with sorafenib and had a rise in the CTC counts and serum AFP levels from the first to the third blood collection. The patient was considered as having SD based on the imaging study. The patient’s CTC count was decreased at the fifth blood collection and was not correlated with the disease status of PD. The patient died within a short period of time after the fifth blood collection. The decrease in the CTC count may reflect the intrinsic characteristics of this patient (Figure 4B). Patient #10 received sorafenib but was unresponsive. The serum AFP levels continued to increase from the first to the fourth blood collection. The CTC count was decreased at the fourth blood collection but remained high throughout the period of study which was consistent with the disease status of PD (Figure 4C). Patient #13 was at SD when enrolled in this study. Serum AFP levels remained high with a slight increase when the disease status changed from SD to PD during the second and third blood collections. The CTC count continued to rise, which was consistent with the change of disease status from SD to PD (Figure 4D). Patient #20 was enrolled in a nivolumab trial. Serum AFP continued to increase. The patient’s CTC count increased at the second blood collection. Treatment was changed to sorafenib and CTC count was decreased to 15 cells/mL after the second blood collection. The patient’s CTC count correlated with the change of disease status (Figure 4E). The initial elevation of the CTC count was likely a pseudo-progression, which is common at the beginning of immunotherapy [24]. Patient #22 received IA-CT and reached SD after the initial treatment. The patient’s serum AFP levels remained high during the same period of time and did not correlate with the patient’s disease status, but the patient’s CTC counts did (Figure 4F). Patient #28 received sorafenib and had an initial serum AFP >100 ng/mL. The patient’s CTC count correlated with the serum AFP level and the patient’s disease status (Figure 4G).

**Figure 5:**
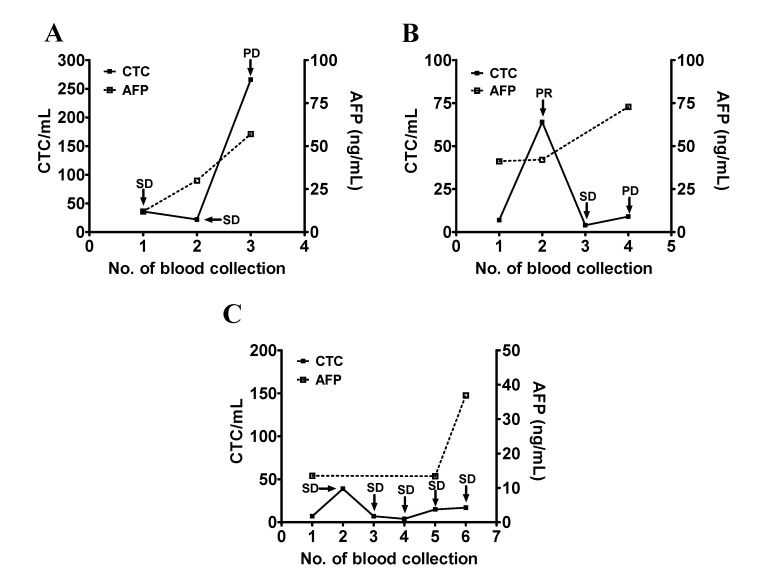
Longitudinal follow-up of CTC counts for monitoring the disease status of patients with HCC (part III). (**A**–**C**) The blood samples were longitudinally collected from patients #15 (panel **A**), #24 (panel **B**), and #25 (panel **C**), who all had a moderate initial serum AFP level followed by an increase. Series testing of the CTC count and the serum AFP level were performed and plotted accordingly. The disease status (SD, PR, and PD) was determined and was used as an indicator of the patient’s clinical outcome.

Patients #15, #24, and #25 had an initial serum AFP <100 ng/mL, which increased during the course of the study. Patient #15 received sorafenib. The patient’s serum AFP level increased slightly which predicted PD at the third blood collection. The change in the CTC count was consistent with the change of disease status from SD to PD (Figure 5A). Patient #24 received sorafenib. The patient’s serum AFP level remained high and the CTC counts correlated with the patient’s disease status (Figure 5B). Patient #25 received sorafenib. Both the serum AFP levels and the CTC counts remained steady throughout the study, which was consistent with the disease status of SD (Figure 5C).

**Figure 6:**
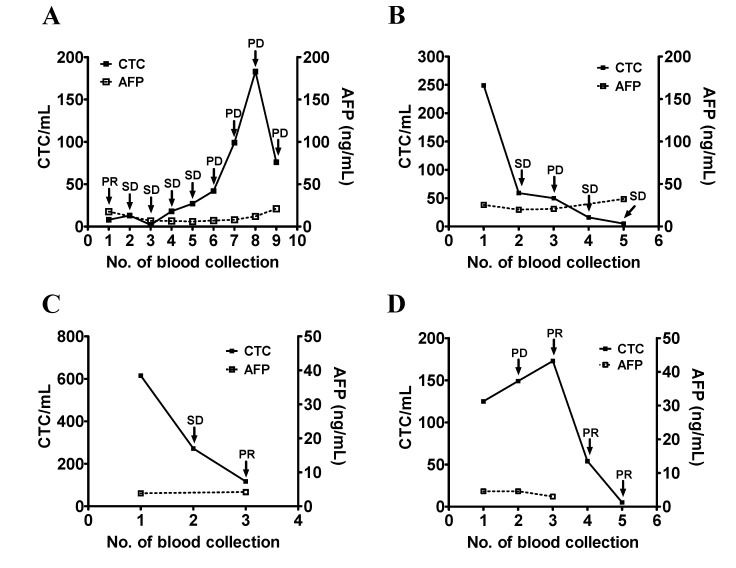
Longitudinal follow-up of the CTC counts for monitoring the disease status of patients with HCC (part IV). (**A**–**D**) The blood samples were longitudinally collected from patients #3 (panel **A**), #23 (panel **B**), #26 (panel **C**), and #27 (panel **D**), who all had a normal serum AFP level throughout the study. Series testing of the CTC count and the serum AFP level were performed and plotted accordingly. The disease status (SD, PR, and PD) was determined and was used as an indicator of the patient’s clinical outcome.

Patients #3, #23, #26, and #27 had an initial serum AFP <100 ng/mL which remained steady during the course of study. Patient #3 was originally treated with sorafenib and after the seventh blood collection was enrolled in a ramucirumab clinical trial. The serum AFP level was within normal range and did not correlate with the change of the patient’s disease status. The CTC counts were mostly consistent with the patient’s disease status (Figure 6A). Patient #23 received sorafenib and was changed to IV-CT after the third blood collection. The serum AFP levels were steady throughout the study. The CTC counts were consistent with the change of the patient’s disease status (Figure 6B). Patient #26 received sorafenib treatment. The serum AFP levels remained low, which did not reflect the change of the patient’s disease status. The patient’s CTC counts decreased and was consistent with the disease status of SD and PR (Figure 6C). Patient #27 received sorafenib during the period of the first and the second blood collection and was enrolled in the clinical trial receiving IA-CT thereafter. The serum AFP levels remained low and did not correlate with the patient’s disease status. The CTC counts were high and were consistent with the disease status of PD at the second blood collection. The number of CTCs gradually decreased and was consistent with the change of disease status (Figure 6D).

## 4. Discussion

Monitoring disease status during treatment is crucial for the clinical management of patients with HCC. In the current study, the number of CTCs and the change in the CTC count are clinically significant and can supplement the use of serum AFP levels for evaluating the disease status of patients with HCC, in particular for the AFP-low cases.

Although serum AFP levels are routinely used in the clinical setting to monitor the disease status of patients with HCC, the serum AFP levels are found in the normal range for up to 42% of patients with HCC [25]. The use of serum AFP levels for HCC surveillance is not recommended by the American Association for the Study of Liver Diseases due to the lack of specificity [26]. Image examinations such as CT or MRI are still the standards for monitoring the treatment response. These procedures either need exposure to radiation or are expensive, and are not performed routinely. Novel HCC biomarkers such as des-gamma-carboxyprothrombin and lectin-bound AFP have been considered for overcoming the drawback of specifically using serum AFP levels for monitoring the treatment response of patients [27]. These markers exhibit poor sensitivity for HCC, particularly in the detection of lesions <3 cm [28]. There is a need for additional markers for monitoring disease status and evaluating the treatment response in patients with HCC, in particular, those patients without elevated serum AFP levels.

With the heterogeneity and complexity of HCC tissue and cancer cells, real-time monitoring of cancer burden in response to therapy is important during the treatment course of patients with HCC. Although imaging study provides a direct assessment of cancer burden on the tissue, the intrinsic property of radiation makes it not suitable for routine longitudinal follow-up of patients. Serum biomarkers such as AFP offer an alternative avenue for real-time monitoring of cancer burden but with clear disadvantages in the clinical setting. Recent studies indicate that liquid biopsies are the suitable biological resources for longitudinal measurements of cancer burden and disease status. Liquid biopsies such as CTC and cell-free circulating tumor DNA offer the option of taking serial samples from a patient to detect changes during disease history and imposed by treatment [29]. Although serial monitoring of cell-free DNA was common, almost all reported CTC studies did only one to two measurements of CTC. For example, CTC counts have been used in previous studies for determining the diagnosis and assessing the prognosis of patients with HCC [15,16,17,30,31,32]. A correlation exists between the postoperative early recurrence of HCC and mesenchymal CTCs [32,33]. An elevated number of CTCs is associated with unfavorable clinicopathologic characteristics responsible for poor prognosis in patients with HCC [34]. An increase in the number of postoperative CTCs when compared to the number of preoperative CTCs is associated with lower survival and higher recurrence among patients with low AFP levels and cirrhosis [35]. Unlike prior studies, in which blood samples were collected at either a single or two time points prior to and after surgery or chemotherapy, blood samples in the current study were collected longitudinally before and during the course of systemic therapies. An evaluation of whether or not the number of CTCs or the change of CTC count related to the change of disease status in HCC patients was based on the CTC counts of each blood collection. With the continuous evolution of intrinsic cellular properties of HCC and the possible changes of therapeutic regimens during the treatment course, longitudinal follow-up of CTCs offers an option for real-time measurement of disease status for patients with HCC.

Several observations were made in the current study. First, CTCs were present in most of the blood samples collected from patients prior to systemic therapies. This is consistent with a number of HCC, papillary thyroid carcinoma, and head and neck cancer studies [19,36,37,38]. Second, patients at PD usually had higher CTC counts compared to patients at SD or PR. Although there is overlap for the two groups of patients, this may have minimal effect on the longitudinal measurements of CTC count and its correlation with patient disease status which is based on comparison to each patient’s baseline CTC count. Further analysis of 85 blood samples from 17 patients with at least three sequential blood collections revealed that a change of the CTC count correlated with a change of disease status in most of the patients. The number of CTC or a change of the CTC count is not in accord with the clinical courses in several patients with high serum AFP, while in other cases AFP provides more useful clinical information and serves as a predictive biomarker even the CTC count reliably follows the clinical course of the patients. According to our analysis of patients who were AFP-low (Figure 6) but underwent disease progression, longitudinal measurements of CTC are informative and provide advantages for monitoring treatment response and disease status of patients without elevated serum AFP.

Simultaneous counting of CTCs and measurement of serum AFP levels are likely to provide more comprehensive information regarding the disease status and treatment response of patients with HCC. This is similar to our previous studies of papillary thyroid carcinoma [36,39]. The presence of CTC compromises serum thyroglobulin (Tg) measurements when the anti-Tg antibody is present in patients with papillary thyroid carcinoma [39]. Combined analyses of CTC counts and serum Tg levels are superior to serum Tg levels alone in establishing the disease status of patients [36]. The combination of CTC counts with serum AFP levels and Tg should decrease false-negative rates and is recommended for disease monitoring of patients with the respective cancer types who are undergoing treatment. Because peripheral blood is easily accessible and provides an important biological resource for diagnosis and monitoring disease progression of cancer patients, combined analyses of CTCs and serum AFP levels with one blood draw can be performed more frequently than imaging and could pave the way for monitoring the disease status and treatment response of HCC patients.

The disease status of several patients was not correlated with the number of CTCs or the change of the CTC counts as discussed in the previous section. The reasons for these observations are not clear. The epithelial–mesenchymal transition (EMT) occurs during cancer progression. A loss or a decrease in EpCAM expression and an increase in mesenchymal markers such as N-cadherin, vimentin, and podoplanin in cancer cells may accompany the EMT [33,40,41]. The number of EpCAM^+^-CTCs is likely to decrease when the disease status of patients changes from SD to PD. This may result in a decrease in EpCAM^+^-CTCs and an increase in EpCAM^−^-CTCs counts when a patient’s cancer is progressing. In the cell filtrate after depletion of CD45^+^ leukocytes, there are EpCAM^−^ cells of which the number is variable in different blood samples from 10^3^ to 10^4^ cells. Usually, less than 5 cells in the cell filtrate were CD45^+^ [18]. Because EpCAM^−^ cells are also present in the healthy control group, it is likely that most of the EpCAM^−^ cells are CD45^−^ circulating endothelial cells or leukocytes such as apoptotic neutrophils [42,43], while some of the cells are EpCAM^−^-CTCs. Future characterization of these EpCAM^−^ cells should provide more insight into the clinical implication of monitoring this cell population. Consistent with this notion, different CTC subpopulations exist and elicit a distinct impact on the disease progression of patients. In this regard, the number of CD133^+^CD54^+^CD44^+^ circulating cancer stem cells is present in the blood and is a biomarker of treatment selection and liver metastasis in patients with CRC [44]. An RNA signature enables high specificity detection of CTCs in HCC [45]. Therefore, it is worthwhile to further characterize different CTC subsets and determine their respective impact on the disease status of HCC patients.

Sequential CTC counts during therapy can supplement serum AFP measurements and is able to provide timely information for monitoring treatment efficacy and clinical outcomes. The findings of the current study may have a significant impact on the clinical management of patients with locally advanced or metastatic HCC, in particular for patients without elevated serum AFP levels.

## Figures and Tables

**Table 1:** Basic characteristics of patients enrolled in this study.

Characteristics	No. of Patients
Total enrollment	30
Stage at the time of diagnosis	
I	8
II	4
III	17
IV	1
Age at enrollment: median (interquartile range)	64 (56–69)
Sex (M/F)	20/10
Hepatitis history	
^1^ HBV^+^ only	11
HCV^+^ only	13
HBV^+^ HCV^+^	2
Non-B, non-C	4
Tumor status at enrollment	
Locally advanced	7
Extrahepatic metastasis	9
Both	14
Treatment at enrollment	
Sorafenib	21
Ramucirumab	2
Nivolumab	1
Thalidomide	2
IA-CT	3
IV-CT	1

^1^ HBV, hepatitis B virus; HCV, hepatitis C virus; IA-CT, intra-arterial chemotherapy; IV-CT, intravenous chemotherapy.

**Table 2:** The testing results for circulating tumor cells (CTCs) and alpha-fetoprotein (AFP) and the disease status of patients with hepatocellular carcinoma (HCC).

Patient ID	Clinical Parameters	Treatment	No. of Blood Collection								
			1	2	3	4	5	6	7	8	9
1	CTCs (cells/mL)	Sorafenib; thalidomide after the 2nd blood collection	4	2	2	10	2	32	12	1	
	AFP (ng/mL)		4737	47,360	−	−	11	<2	<2	<2	
	Disease status		−	^1^ SD	PR	PR	PR	PR	PR	PR	
2	CTCs (cells/mL)	Sorafenib	45	2	3	26	26	43			
	AFP (ng/mL)		323	675	3356	4822	4171	−			
	Disease status		−	SD	SD	PD	PD	PD			
3	CTCs (cells/mL)	Sorafenib; ramucirumab after the 7th blood collection	8	13	2	18	27	42	99	183	76
	AFP (ng/mL)		18	−	7	7	6	7	8	12	21
	Disease status		PR	SD	SD	SD	SD	PD	PD	PD	PD
4	CTCs (cells/mL)	Sorafenib	21	66	179	129	8				
	AFP (ng/mL)		5222	12,929	16,727	−	−				
	Disease status		−	SD	SD	PD	PD				
5	CTCs (cells/mL)	Sorafenib	20	211							
	AFP (ng/mL)		59,233	−							
	Disease status		−	PD							
6	CTCs (cells/mL)	Sorafenib; thalidomide after the 2nd blood collection	73	25							
	AFP (ng/mL)		35	−							
	Disease status		−	PD							
7	CTCs (cells/mL)	Sorafenib	64								
	AFP (ng/mL)		542								
	Disease status		PD								
8	CTCs (cells/mL)	Sorafenib	88								
	AFP (ng/mL)		8535								
	Disease status		PD								
9	CTCs (cells/mL)	Sorafenib	43								
	AFP (ng/mL)		656								
	Disease status		PD								
10	CTCs (cells/mL)	Sorafenib	74	94	1148	315					
	AFP (ng/mL)		146	−	231	543					
	Disease status		−	PD	PD	PD					
11	CTCs (cells/mL)	Sorafenib	9	19							
	AFP (ng/mL)		4	2							
	Disease status		PD	−							
12	CTCs (cells/mL)	Sorafenib	198	18							
	AFP (ng/mL)		12,222	26,771							
	Disease status		−	SD							
13	CTCs (cells/mL)	Sorafenib	42	615	902						
	AFP (ng/mL)		421	390	596						
	Disease status		SD	SD	PD						
14	CTCs (cells/mL)	Sorafenib	109								
	AFP (ng/mL)		54								
	Disease status		PD								
15	CTCs (cells/mL)	Sorafenib	36	22	266						
	AFP (ng/mL)		12	30	57						
	Disease status		−	SD	PD						
16	CTCs (cells/mL)	IA-CT	13	16	5						
	AFP (ng/mL)		27,621	−	−						
	Disease status		PD	−	PD						
17	CTCs (cells/mL)	IA-CT	100	10	42	29	34	98			
	AFP (ng/mL)		>500,000	>80,000	28,614	23,990	35,835	−			
	Disease status		PD	PD	PD	PD	PD	PD			
18	CTCs (cells/mL)	IV-CT	2	12							
	AFP (ng/mL)		6633	11,677							
	Disease status		−	PD							
19	CTCs (cells/mL)	Ramucirumab	6	5	4	21	3				
	AFP (ng/mL)		840	11	11	3	−				
	Disease status		−	PR	PR	PR	PR				
20	CTCs (cells/mL)	Nivolumab; sorafenib after the 2nd blood collection	12	310	15						
	AFP (ng/mL)		2491	3513	4508						
	Disease status		−	PD	PR						
21	CTCs (cells/mL)	Ramucirumab	6	16							
	AFP (ng/mL)		14	14							
	Disease status		−	PD							
22	CTCs (cells/mL)	IA-CT	139	15	7						
	AFP (ng/mL)		6453	9695	−						
	Disease status		−	SD	SD						
23	CTCs (cells/mL)	Sorafenib; IA-VT after the 3rd blood collection	249	59	50	16	5				
	AFP (ng/mL)		25	20	21	−	32				
	Disease status			SD	PD	−	SD				
24	CTCs (cells/mL)	Sorafenib	7	64	4	9					
	AFP (ng/mL)		41	42	-	73					
	Disease status		−	PR	SD	PD					
25	CTCs (cells/mL)	Sorafenib	7	39	7	4	15	17			
	AFP (ng/mL)		14	−	−	−	13	37			
	Disease status		−	SD	SD	SD	SD	SD			
26	CTCs (cells/mL)	Sorafenib	615	272	117						
	AFP (ng/mL)		4	−	4						
	Disease status		−	SD	PR						
27	CTCs (cells/mL)	Sorafenib; IA-CT after the 2nd blood collection	125	149	173	54	5				
	AFP (ng/mL)		5	4	3	−	−				
	Disease status		−	PD	PR	PR	PR				
28	CTCs (cells/mL)	Sorafenib	16	18	1955	165					
	AFP (ng/mL)		1254	1188	1859	689					
	Disease status		−	PD	PD	PR					
29	CTCs (cells/mL)	Thalidomide	134	24							
	AFP (ng/mL)		16	29							
	Disease status		−	PD							
30	CTCs (cells/mL)	Thalidomide	15								
	AFP (ng/mL)		67,658								
	Disease status		SD								

^1^ SD, stable disease; PR, partial response; PD, progressive disease.
